# Monitoring of Antimicrobial Susceptibility of Bacteria Isolated from Poultry Farms from 2014 to 2018

**DOI:** 10.1155/2021/6739220

**Published:** 2021-09-10

**Authors:** Engy Ahmed Hamed, May Fathy Abdelaty, Hend Karam Sorour, Heba Roshdy, Mona Aly Abdelhalim AbdelRahman, Ola Magdy, Waleed Abdelfatah. Ibrahim, Ahmed Sayed, Hytham Mohamed, Mohammed Iraqi Youssef, Wafaa Mohamed Hassan, Heba Badr

**Affiliations:** Reference Laboratory for Veterinary Quality Control on Poultry Production, Animal Health Research Institute, Agricultural Research Center (ARC), Nadi El-Seid Street, Dokki P.O. Box 246, Giza 12618, Egypt

## Abstract

The current situation of antibiotic resistance of most bacterial pathogens was a threat to the poultry and public health with increasing economic losses. Regarding this problem, monitoring of the circulating microorganisms occurred with the antibiotic resistance profile. A total of 657 different samples from internal organs (liver, heart, lung, and yolk) and paper-lining chick boxes were collected from native chicken farms which were submitted to the Reference Laboratory for Veterinary Quality Control on Poultry Production in the period from 2014 to 2018 for the detection of *Salmonella*, *Escherichia coli* (*E*. *coli)*, and *Staphylococcus*. The bacterial isolates were tested for their antimicrobial susceptibility by disk diffusion technique. *Salmonella* was isolated from 128 out of 657 (19.5%), *E*. *coli* was isolated from 496 out of 657 (75.5%), and *Staphylococcus* species was isolated from 497 out of 657 (75.6%). All *Salmonella* positive samples were examined for antibiotic resistance against 10 different antibiotics, and the highest percentage all over the five years was against penicillin, ampicillin, and tetracycline. All E. *coli* positive samples were examined for antibiotic resistance against 14 different antibiotics, and the highest percentage all over the five years was with ampicillin, tetracycline, norfloxacin, streptomycin, and danofloxacin. All *Staphylococcus* positive sample species were examined for antibiotic resistance against 14 different antibiotics, and the highest percentage of resistance all over the five years was shown with tetracycline, streptomycin, ampicillin, and nalidixic acid.

## 1. Introduction

Poultry is one of the major sources of protein for humans, and in last year, there were new breeds that could reach the growth weight in a short period maybe six weeks or less. Therefore, when human eats this poultry meat, it will affect the normal microflora in human intestinal tract and also may lead to development of resistance to these antibiotics in the human body [1].

There are many mechanisms of generation of antibiotic resistance genes in bacteria. First one occurred in producer bacteria which generate self-resistant genes to protect themselves from their own antibiotics; this mechanism is called the self-defense mechanism. Misuse of antibiotics in treatment or as growth promoters is one of the important causes of mutation in genes of pathogenic bacteria, and the generation of new strains of resistance bacteria to these antibiotics may also have an effect on commensal bacteria which present normally in the gastrointestinal tract and produce antibiotic resistance genes which are transferred to the pathogenic bacteria by plasmids through (horizontal gene transfer) which lead to a generation of a new pathogenic antibiotics resistant strain. Another way for the production of antibiotic bacterial resistant strains occurs outside the host body (in the soil) via horizontal gene transfer through transferring the resistance gene from the producer bacteria or from the nonproducer bacteria (environmental bacteria) to pathogenic strains through the plasmid or phage [2]. Antibiotic-resistant bacteria in poultry farms lead to significant economic losses by spending a lot of money on the treatment and disinfection of poultry houses after infection. Therefore, you must stop abusing antibiotics, and use antibiotics as a treatment only after performing an antibiotic sensitivity test in vitro to determine effective antibiotic therapy, and give antibiotics in the recommended dose, period, and follow the withdrawal period list for preventing its effect on human health [3].

Salmonellosis, colibacillosis, and staphylococcosis are major diseases that affect poultry farms and cause a high economic loss due to high mortality and morbidity rates, cause drop in egg production, and affect in feed conversion and growth rates. All of them cause septicemic lesions, and we can differentiate between them through postmortem inspection. *Salmonella* causes unabsorbed yolk sac in young chicks and focal necrosis in the mucosa of the small intestine, and sometimes, with caseated material in cecal cores, it affects oviducts and ovaries in adult chickens. *E*. *coli* causes omphalitis in young chicks and salpingitis, and the respiratory form causes the swollen head syndrome and airsacculitis. *Staphylococcus* causes osteomyelitis and arthritis [4–6].

In developing countries, the farmers use antimicrobials in subtherapeutic doses for prophylaxis or as growth promoters in poultry farms. All the aforementioned cases of antibiotic misuse affect bacterial response to these antibiotics as treatment and development of multidrug-resistant (MDR) bacteria which may be transmitted to humans indirectly by the consumption of poultry or poultry byproducts [7, 8]. The aim of this study is to monitor the antibiotic resistance profile of *Salmonella*, *E*. *coli*, and *Staphylococcus* isolated from poultry farms during the period from 2014 to 2018.

## 2. Materials and Methods

### 2.1. Sampling

A total of 657 different samples from internal organs (liver, heart, lung, and yolk) and paper-lining chick boxes were collected from native chicken farms which were submitted to the Reference Laboratory for Veterinary Quality Control on Poultry Production (RLQP) from 2014 to 2018.

This study was done on different ages including apparently healthy and diseased chickens which showed, in postmortem examination, different criteria such as congestion, necrosis in the liver, bronzy liver, congestion in the heart, congestion in the lung, pneumonia, pericarditis, perihepatitis, omphalitis, typhlitis, and enlarged ceca.

Pooled organ samples were collected such as the liver, heart, and lung, and on the other side, the yolk sac and paper-lining chick boxes or two ceca were collected separately under aseptic conditions according to Middleton et al. [9]. All samples were examined bacteriologically for the presence of *Salmonella*, *E. coli*, and *Staphylococcus*.

### 2.2. Bacteriological Examination

 
*Salmonella* isolation and identification were done according to standard methods [10, 11]
 
*E*. *coli* isolation was carried according to Lee et al. [12]
 
*Staphylococcus* isolation was done according to standard methods [13]

### 2.3. Antimicrobial Sensitivity Test

The antimicrobial sensitivity test of all isolated *Salmonella*, *E. coli*, and *Staphylococcus* strains was conducted according to Koneman et al. [14] by the disc diffusion method, and different antibiotic discs were used such as ampicillin Amp10, chloramphenicol C30, ciprofloxacin CIP5, colistin sulfate CT25, danofloxacin DX, doxycycline Do30, enrofloxacin ENR5, erythromycin E15, fosfomycin FOS-200, levofloxacin LEV-5, nalidixic acid NA30, norfloxacin NOR10, nitrofurantoin F300, penicillin P10, streptomycin S10, tetracycline T30, and trimethoprim-sulfamethoxazole 25 SXT; Oxoid. The results were interpreted according to the Clinical and Laboratory Standards Institute [15].

## 3. Results

### 3.1. Postmortem Inspection Results

*Salmonella*-suspected samples showed congestion and enlarged liver and spleen were enlarged, the two cecal cores were enlarged and contained caseated material, oviduct and ovaries were affected, fibrinous-perihepatitis, fibrinous-pericarditis, the kidney was congested and enlarged, and unabsorbed yolk sac was found in young chicks. *E. coli*-suspected samples showed omphalitis in newly hatched chicks, and the other ages had airsacculitis with turbidity in the air sac, enlarged liver and heart with fibrinous pericarditis and fibrinous perihepatitis, and congestion in the lung; in some cases, swollen head and salpingitis were found. *Staphylococcus*-suspected samples showed arthritis, swollen joints with a presence of yellow caseated material, femur head which may be separated from its shaft, and congestion in the internal organs; in some cases, the liver was enlarged and contained abscess.

### 3.2. Isolation, Identification, and Antibiotic Resistance

**Figure 1:**
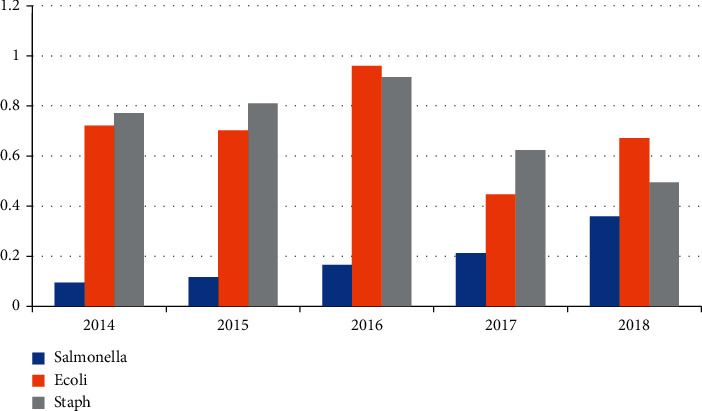
Incidence of *Salmonella*, *E*. *coli*, and *Staphylococcus* isolation from 2014 to 2018.

**Figure 2:**
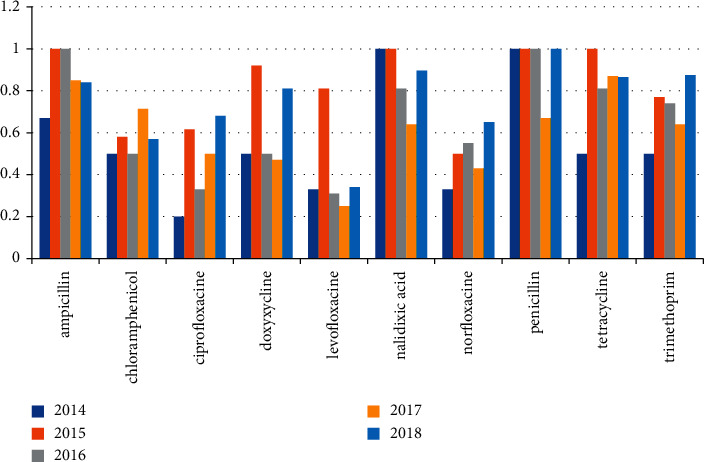
Antibiotic resistance profile of *Salmonella* isolates from 2014 to 2018.

*Salmonella* was isolated from 128 out of 657 (19.5%) examined farms which were examined in the period from 2014 to 2018.  Table 1 and Figure 1 mention the number of positive farms which were examined every year. All *Salmonella* isolates were examined for antibiotic resistance against 10 different antibiotics (ampicillin, chloramphenicol, ciprofloxacin, doxycycline, levofloxacin, nalidixic acid, norfloxacin, penicillin, tetracycline, and trimethoprim). We found that the highest percentage of resistance was with penicillin and then ampicillin. All over the years, the percentage of resistance was high with chloramphenicol, ciprofloxacin, norfloxacin, tetracycline, and trimethoprim. The percentage of resistance against antibiotics is mentioned individually in Table 2 and Figure 2.

**Figure 3:**
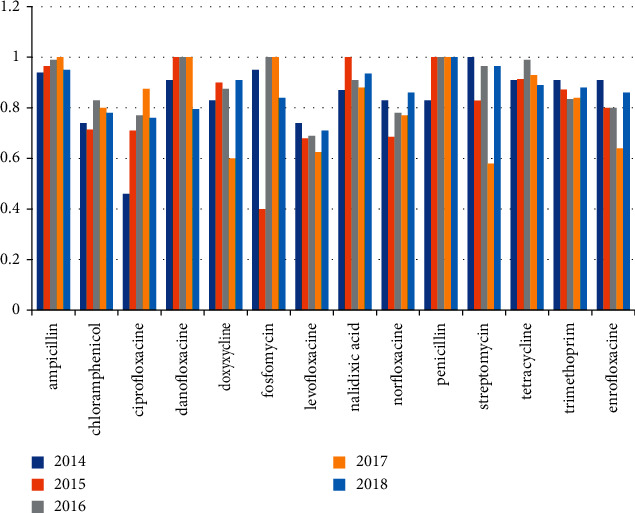
Antibiotic resistance profile of *E*. *coli* isolates from 2014 to 2018.

*E. coli* was isolated from 496 out of 657 (75.5%) examined farms which were examined in the period from 2014 to 2018.  Table 1 and Figure 1 mention the number of positive farms every year. Over the period from 2014 to 2018, all positive isolates of *E. coli* were examined for antibiotic resistance against 14 different antibiotics (ampicillin, chloramphenicol, ciprofloxacin, danofloxacin, doxycycline, fosfomycin, levofloxacin, nalidixic acid, norfloxacin, penicillin, tetracycline, streptomycin, trimethoprim, and enrofloxacin). The highest percentage of resistance all over the years was observed in ampicillin, penicillin, tetracycline, nalidixic acid, streptomycin, and trimethoprim, followed by fosfomycin, doxycycline, and also danofloxacin. The percentage of resistance against antibiotics is mentioned individually in Table 3 and Figure 3.

**Figure 4:**
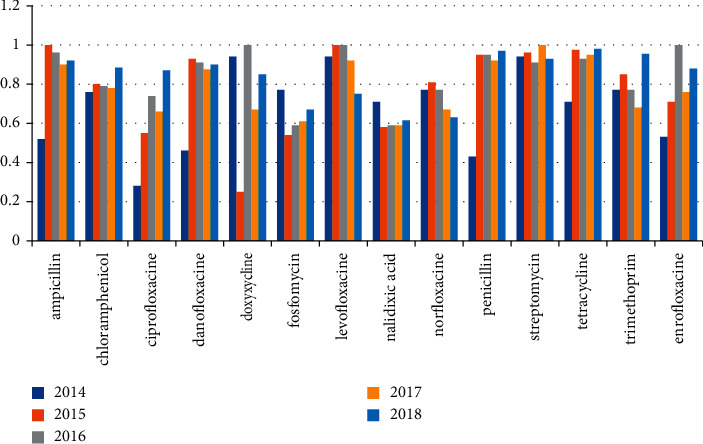
Antibiotic resistance profile of *Staphylococcus* isolates from 2014 to 2018.

*Staphylococcus* was isolated from 497 out of 657 (75.6%) examined farms which were examined in the period from 2014 to 2018.  Table 1 and Figure 1 mention the number of positive farms isolated every year. Over the period from 2014 to 2018, all positive isolates for *Staphylococcus* species were examined for antibiotic resistance against 14 different antibiotics (ampicillin, chloramphenicol, ciprofloxacin, doxycycline, fosfomycin, levofloxacin, nalidixic acid, norfloxacin, nitrofurantoin, penicillin, streptomycin, tetracycline, trimethoprim, and enrofloxacin). The highest percentage of resistance all over the years was observed in tetracycline, streptomycin, ampicillin, and nalidixic acid followed by penicillin, doxycycline, fosfomycin, trimethoprim, and chloramphenicol. The percentage of resistance against antibiotics is mentioned individually in Table 4 and Figure 4.

## 4. Discussion

The major problem nowadays all over the world in poultry production is infection with multidrug-resistant bacteria. The resistance to existing antimicrobials is widespread and of concern to poultry veterinarians because the administration of antimicrobials to chickens as therapeutic and subtherapeutic levels has been an integral part of poultry production. The practice of using unsusceptible antibiotics to the bacteria strain or using the antibiotics in low doses as growth promoters in poultry dietary plays a role in encouraging antibiotic-resistant organisms. Once established, resistant organisms can spread from farms to humans through the consumption of contaminated food [16]. In this study, we monitor some antibiotics and their effect on three major bacterial strains (*Salmonella*, *E. coli*, and *Staphylococcus*) which affect our poultry farms in the period from 2014 to 2018. Salmonellosis is one of the major bacterial diseases which affects the productivity of poultry farms, and we found an increase of *Salmonella* infection over the five years; also, Witkowska et al. [17] found an increase of *Salmonella* infection in 2015 and 2016 poultry farms. Also, *Salmonella*, *E. coli*, and *Staphylococcus* play a role in human foodborne illness due to the consumption of poultry infected with these microorganisms if they contain antibiotic resistance genes or not [18–20].

### 4.1. *Salmonella* Species Isolates

In this study, *Salmonella* was isolated from 128 out of 657 (19.5%) examined poultry farms in the period from 2014 to 2018, and these results were nearly average percentage to what Ammar et al. [21, 22] observed, who found *Salmonella* in examined poultry farms in Egypt in their study by 17% and 16%, respectively. On the contrary, Badr et al. and Shehata et al. [18, 23] found *Salmonella* in low percentage, about 7% and 7.1%, respectively, in samples isolated from poultry farms in Egypt. Moreover, El-Sharkawy et al. [24] isolated *Salmonella* in high percentage (41%) from poultry farms in Egypt; also, *Salmonella* was isolated from poultry farms in Brazil and USA in high percentage, about 37% and 56%, respectively [25, 26]. In our study, in the period from 2014 to 2018, all positive samples for *Salmonella* were examined for antibiotic resistance against 10 different antibiotics which showed that the highest average percentage of resistance was with penicillin and ampicillin followed by tetracycline, nalidixic acid, trimethoprim, doxycycline, and chloramphenicol (94.4, 90.6, 86.2, 82.5, 78.2, 70.7, and 62.2%, respectively). Subsequently, the percentage of resistance was decreased in norfloxacin, ciprofloxacin, and levofloxacin (53.2, 52.3, and 39.4%, respectively). Also, Egypt [23] recorded that most of the tested *Salmonella serovars* were multidrug resistant and had high MAR indicated against the commonly used antibiotics in the poultry industry in Egypt. The data showed that 94.4%, 72.2%, 44.4%, 44.4%, 33.3%, and 33.3% of the tested *Salmonellae* were resistant to ampicillin, sulphamethoxazole/trimethoprim, tetracycline, chloramphenicol, doxycycline, and norfloxacin, respectively. Moreover, Hassan et al. [27] revealed that *Salmonella* isolates showed complete resistance against penicillin, while they were highly resistant to nalidixic acid (80.8%), sulphamethoxazole/trimethoprim (76.9%), ampicillin (69.2%), and oxytetracycline (65.4%), while Eguale [28] reported that 42.3% were resistant to ampicillin and chloramphenicol, while 30.9%, 19%, 7.7%, and 3.9% were resistant to tetracycline, nalidixic acid, ciprofloxacin, and trimethoprim for *Salmonella* isolated from broilers in Ethiopia. In contrast, Wang et al. [26], in USA, mentioned the rates of isolates resistant to ciprofloxacin and tetracycline (100%), then chloramphenicol (99%), ampicillin (97%), and trimethoprim-sulfamethoxazole (97%).

### 4.2. *E. coli* Isolates

In our study, we detected *E. coli* in 496 out of 657 (75.5%) poultry farms examined in the period from 2014 to 2018; these chickens suffered from colibacillosis, and symptoms perihepatitis, pericarditis, airsacculitis, and omphalitis were found in postmortem examination; these results were near to what Stella et al. and Davis et al. [29, 30] observed who found that the percentage of *E. coli* was high, about 60% and 88% of examined chicken samples in Brazil and the USA, respectively, while Ameen-Ur-Rashid et al. and Ibrahim et al. [31, 32] found *E. coli* in low percentage, about 35.3% and 34% of examined chicken samples in Pakistan and Jordan, respectively. All *E. coli* strains isolated in the period from 2014 to 2018 were examined for antibiotic resistance against 14 different antibiotics and showed that there was high resistance all over the five years which ranged from 97.3% to 63.5%, and these results agreed with Ibrahim et al., Jahantigh et al., and Rafiqueet et al. [33–35] who found the highest percentage of resistance to ampicillin, ciprofloxacin, doxycycline, nalidixic acid, streptomycin, tetracycline, and trimethoprim in Egypt, Iran, and Pakistan, respectively, and disagreed with what Ameen-Ur-Rashid et al. and Ibrahim et al. [31, 33] observed who examined the resistance of *E. coli* to the antibiotics and found low percentages to ampicillin, ciprofloxacin, chloramphenicol, doxycycline, enrofloxacin, norfloxacin, levofloxacin, and trimethoprim which ranged from 9% to 50% in Pakistan and Egypt, respectively.

### 4.3. *Staphylococcus* Isolates

In this study, *Staphylococcus* species were detected in 497 out of 567 (75.6%) examined poultry farms in the period from 2014 to 2018, and the samples taken from these farms mostly suffered from affected joints, congestion in internal organs, and abscess in the liver, and these results were nearly the same to what Mariam-Shokery et al. [36] observed who found *Staphylococcus* species in 69.23% of infected examined chicken farms, while Amen et al. [20] found *Staphylococcus* species in 24.75% of examined chicken farms. All *Staphylococcus* isolates in the period from 2014 to 2018 were examined for antibiotic resistance against 14 antibiotics, and our results reported high resistance against tetracycline, streptomycin, ampicillin, and nalidixic acid, followed by penicillin, doxycycline, trimethoprim, and chloramphenicol all over the five years, and these results were the same to those mentioned in studies of other countries such as Indonesia, Pakistan, South Africa, and Nigeria [8, 37–39], respectively, while the results of tetracycline and chloramphenicol detected in [8] and the results of ampicillin, ciprofloxacin, enrofloxacin, and streptomycin examined in [20] were lower than what we found in our study.

## 5. Conclusions

This result indicates that the *Salmonella* infection in poultry has been dramatically increased annually with severe economic loss. It may be due to focusing on viral diseases such as AI (avian influenza), especially after AI outbreak in 2006 in Egypt at the expense of bacterial diseases in poultry.

Year 2016 shows a higher incidence of isolation of *E. coli* and *Staphylococcus* from poultry farms than in other years.

This study shows an increase in the percentage of resistance in some antibiotics such as levofloxacin, norfloxacin, doxycycline, penicillin, tetracycline, and trimethoprim during the period from 2014 to 2018 in examined microbes (*Salmonella*, *E. coli*, and *Staphylococcus*), while still, *Salmonella* strains have moderate resistance to quinolones all over the five years. This increasing percentage of these antibiotics indicates the negative feedback of misuse of these antibiotics in poultry farms which act as a mirror of this misusage.

Misuse of antibiotics as unspecific treatment or as growth promoters may enhance generations of antibiotic resistance genes in bacteria which may infect poultry farms and may lead to high cost of treatment of infected farms due to the lack of antibiotics that can be used to treat multidrug-resistant bacteria, as well as it can affect human health.

We recommend not to use antibiotics as growth promoters, must make antibiotic sensitivity test for infected farms before giving any antibiotics as treatment, and must take in our mind the withdrawal time of used antibiotics to protect human health. We must look for other ways to treat the microbial infection such as the use of natural products which have antimicrobial activity like essential oils, and the implementation of biosafety and biosecurity measures in poultry farms.

## Figures and Tables

**Table 1:** Incidence of *Salmonella*, *E*. *coli*, and *Staphylococcus* isolated from poultry farms in the period 2014 to 2018.

	*Salmonella*		*E*. *coli*		*Staphylococcus*	
	No. of positive samples	%	No. of positive samples	%	No. of positive samples	%
2014 (*n* = 134)	13	9.7	99	73.9	106	79.1
2015 (*n* = 118)	14	11.9	85	72	98	83
2016 (*n* = 178)	30	16.9	175	98.3	167	93.8
2017 (*n* = 83)	18	21.7	38	45.8	53	63.9
2018 (*n* = 144)	53	36.8	99	68.8	73	50.7
Total (*n* = 657)	128	19.5	496	75.5	497	75.6

*n* = total number of examined farms.

**Table 2:** Antibiotic resistance profile of *Salmonella* isolates from 2014 to 2018.

Antibiotics	2014 (*n* = 13)	2015 (*n* = 14)	2016 (*n* = 30)	2017 (*n* = 18)	2018 (*n* = 53)	Total (*n* = 128)
Ampicillin	67%	100%	100%	85%	84%	90.6%
Chloramphenicol	50%	58%	50%	71.4%	57%	62.2%
Ciprofloxacine	20%	61.5%	33%	50%	68%	52.3%
Doxycycline	50%	92%	50%	47%	81%	70.7%
Levofloxacine	33%	81%	31%	25%	34%	39.4%
Nalidixic acid	100%	100%	81%	64%	89.5%	82.5%
Norfloxacine	33%	50%	55%	43%	65%	53.2%
Penicillin	100%	100%	100%	67%	100%	94.4%
Tetracycline	50%	100%	81%	87%	86.5%	86.2%
Trimethoprime	50%	77%	74%	64%	87.5%	78.2%

Total = total percentage of resistant strains all over five years; *n* = number of positive samples.

**Table 3:** Antibiotic resistance profile of *E*. *coli* isolates from 2014 to 2018.

Antibiotics	2014 (*n* = 99)	2015 (*n* = 85)	2016 (*n* = 175)	2017 (*n* = 38)	2018 (*n* = 99)	Total (*n* = 496)
Ampicillin	94%	96.5%	99%	100%	95%	97.2%
Chloramphenicol	74%	71.4%	83%	80%	78%	78.4%
Ciprofloxacine	46%	71%	77%	87.5%	76%	71%
Danofloxacin	91%	100%	100%	100%	79.5%	84.3%
Doxycycline	83%	90%	87.5%	60%	91%	85.5%
Fosfomycine	95%	40%	100%	100%	84%	85.5%
Levofloxacine	74%	68%	69%	62.5%	71%	69.7%
Nalidixic acid	87%	100%	91%	88%	93.5%	92%
Norfloxacine	83%	68.5%	78%	77%	86%	77.3%
Penicillin	83%	100%	100%	100%	100%	95%
Streptomycin	100%	82.8%	96.5%	58%	96.5%	89.6%
Tetracycline	91%	91.4%	99%	93%	89%	93.4%
Trimethoprime	91%	87.2%	83.5%	84%	88%	86%
Enrofloxacine	91%	80%	80%	64%	86%	82.4%

Total = total percentage of resistant strains all over five years; *n* = number of positive samples.

**Table 4:** Antibiotic resistance profile of *Staphylococcus* isolates from 2014 to 2018.

Antibiotics	2014 (*n* = 106)	2015 (*n* = 98)	2016 (*n* = 167)	2017 (*n* = 53)	2018 (*n* = 73)	Total (*n* = 497)
Ampicillin	52%	100%	96%	90%	92%	91.6%
Chloramphenicol	76%	80%	79%	78%	88.4%	80.6%
Ciprofloxacine	28%	55%	74%	66%	87%	60.4%
Doxycycline	46%	93%	91%	87.5%	90%	85%
Fosfomycine	94%	25%	100%	67%	85%	83.1%
Levofloxacine	77%	54%	59%	61%	67%	60.8%
Nalidixic acid	94%	100%	100%	92%	75%	90.3%
Norfloxacine	71%	58%	59%	59%	61.5%	60%
Nitroforantine	77%	81%	77%	67%	63%	74.1%
Penicillin	43%	95%	95%	92%	97%	87%
Streptomycin	94%	96%	91%	100%	93%	92.9%
Tetracycline	71%	97.5%	93%	95%	98%	93%
Trimethoprim	77%	85%	77%	68%	95.5%	81.3%
Enrofloxacine	53%	71%	100%	76%	88%	72.7%

Total = total percentage of resistant strains all over five years; *n* = number of positive samples.

## Data Availability

Samples were submitted to the Reference Laboratory for Veterinary Quality Control on Poultry Production for bacterial diseases' examination and sensitivity test for specific antibiotics.
